# A Case Report on Ambiguous Genitalia: A Diagnostic, Therapeutic, and Cosmetic Challenge

**DOI:** 10.7759/cureus.41142

**Published:** 2023-06-29

**Authors:** Mukta Agarwal, Shivangni Sinha, Sarsij Sharma, Smita Singh, Siddhant Roy

**Affiliations:** 1 Obstetrics and Gynaecology, AII India Institute of Medical Sciences, Patna, Patna, IND; 2 Obstetrics and Gynaecology, All India Institute of Medical Sciences, Patna, Patna, IND; 3 Burns and Plastic Surgery, All India Institute of Medical Sciences, Patna, Patna, IND; 4 Obstetrics and Gynecology, AII India Institute of Medical Sciences, Patna, Patna, IND; 5 Urology, All India Institute of Medical Sciences, Raipur, Raipur, IND

**Keywords:** ambiguous genitalia, aesthetic surgery, prophylactic gonadectomy, genitoplasty, : partial androgen insensitivity syndrome

## Abstract

Ambiguous genitalia is a matter of concern and needs thorough evaluation and treatment. Gonadectomy becomes a potentially lifesaving procedure in patients with partial androgen insensitivity due to the increased risk of malignancy if left undiagnosed.

We present a case report of two patients in their late 20s and 30s, raised as girls, who came with complaints of primary amenorrhea with ambiguous genitalia. Both patients had features of masculinization. Her MRI revealed an absent uterus, cervix, upper 2/3 of the vagina, and ovaries, with the presence of bilateral testicles. She was diagnosed with partial androgen insensitivity syndrome. The first patient underwent bilateral gonadectomy with hernia repair and nerve-sparing reduction clitoroplasty with labioplasty. She is under close follow-up with a further plan for augmentation mammoplasty. The second patient, however, refused clitoroplasty and underwent bilateral gonadectomy.

Androgen insensitivity syndrome is an X-linked inheritance with a mutation in the AR gene. It consists of a spectrum of conditions ranging from complete insensitivity to less insensitivity towards testosterone, which results in a complete, partial, and mild form of androgen insensitivity syndrome. Studies have been done on cosmetic outcomes after genitoplasty in children with genital atypicalities, which showed significant improvement (p<0.001) and no difference in ratings by parents and surgeons.

Surgeries done on patients with partial androgen insensitivity syndrome are not only lifesaving procedures, but with reasonable reassurance, these aesthetic surgeries help people live a life that otherwise would have been genetically compromised.

## Introduction

Living with a rare disease is itself a challenge and a continuing stigma in this present world. Ambiguous genitalia belongs to one such spectrum with a varied presentation, making its diagnosis difficult. It also stands as a therapeutic dilemma and a challenge to clinicians. It is a congenital condition caused by atypical development in chromosomal, gonadal, and phenotypic sex expression. External sex organs may differ from internal sex organs or genetic sex. It is a sexual development condition. Usually, ambiguous genitalia is visible at or shortly after birth, which can be quite upsetting for families. The sex organs of men and women develop from the same tissue. The presence or absence of male hormones determines whether this tissue develops into male or female organs. In a genetically male fetus, a lack or shortage of male hormones can result in ambiguous genitalia, whereas exposure to male hormones during development results in ambiguous genitalia in a genetically female fetus. Other reasons are genetic mutation, chromosomal abnormalities, and enzymatic deficiency. Family history may be a factor in the development of ambiguous genitalia because many sex development disorders are the result of inherited genetic anomalies.

We present a case report of two patients raised as girls with features of masculinization, primary amenorrhea, and ambiguous genitalia.

## Case presentation

Case report 1

A 20-year-old woman presented at our OBG Outpatient Department three months ago with complaints of primary amenorrhea and a lump palpable in the inguinal area. She was seventh in birth order and had no notable family background. Patients and family members had previously failed to seek medical guidance.

On examination, her height was 161 cm, her weight was 65 kg, and her BMI was 25.07 kg/m^2^. She had a male-type body contour with hirsutism (Ferriman-Gallwey Score: 19). Her breast tissues were not developed (Tanner’s stage 2), and axillary hair was present with pubic hair developed to Tanner’s stage 4. In the abdomen, bilateral lumps were palpable in the inguinal region. On local examination, there was clitoromegaly (6 cm on the phall-o-meter), and a urethral opening 1 cm below the clitoris was seen. Both labia majora and minora were well formed, with a blind vagina of 3 cm. Per-rectally, the uterus was not palpable.

Her testosterone levels were high: 196.6 ng/dL (typical adult male 200-800 ng/dL, adult female 20-80 ng/dL). Her serum LH (34.2 mIU/ml; normal range: 21.9-56.6 IU/mL) and FSH (25.9 mIU/ml; normal range: 4-25 IU/L) levels were elevated, but she had a low level of estradiol (40.8 pg/mL; normal range: 30-400 pg/mL). However, the levels of dehydroepiandrosterone sulfate (DHEAS; 166.4 g/dL, normal range: 145-395), androstenedione (1.05 ng/mL, normal range: 0.7-3.1 ng/mL), 17-hydroxyprogesterone (0.60 ng/mL, normal range: 2.8 ng/mL), and cortisol (14.6 mcg/dL; normal range: 6-23 mcg/dL) were within the standard range.

An MRI revealed that there was no uterus, cervix, upper 2/3rd vagina, or ovaries, but there were bilateral testicles with seminal vesicles, vas deference, and a little prostrate-like tissue at the bladder base. There were no numerical or structural irregularities in the karyotype, which was 46XY.

**Figure 1:**
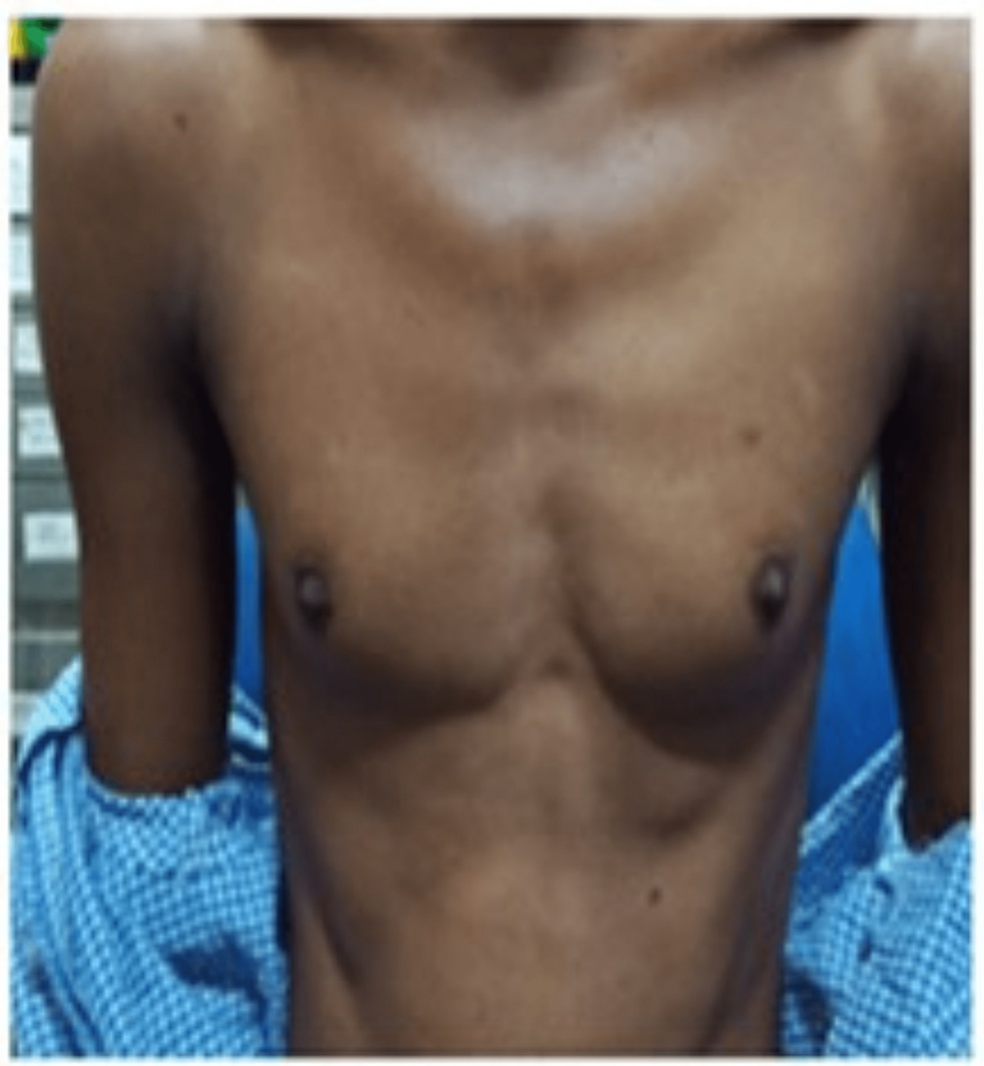
The physical characteristics of the patient illustrate the male type of body contour. Self-ownership declared.

**Figure 2:**
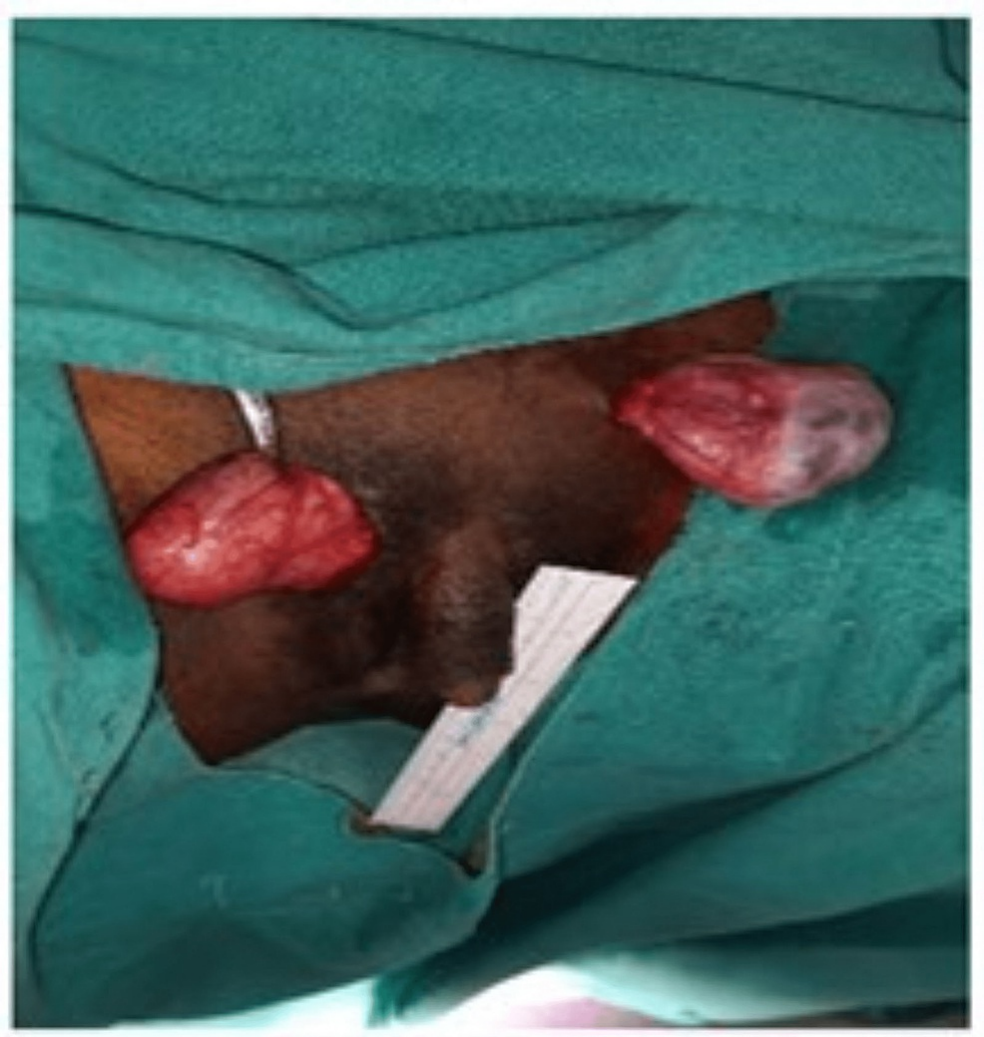
Per-operative findings suggestive of bilateral gonads and clitoromegaly. Self-ownership declared.

**Figure 3:**
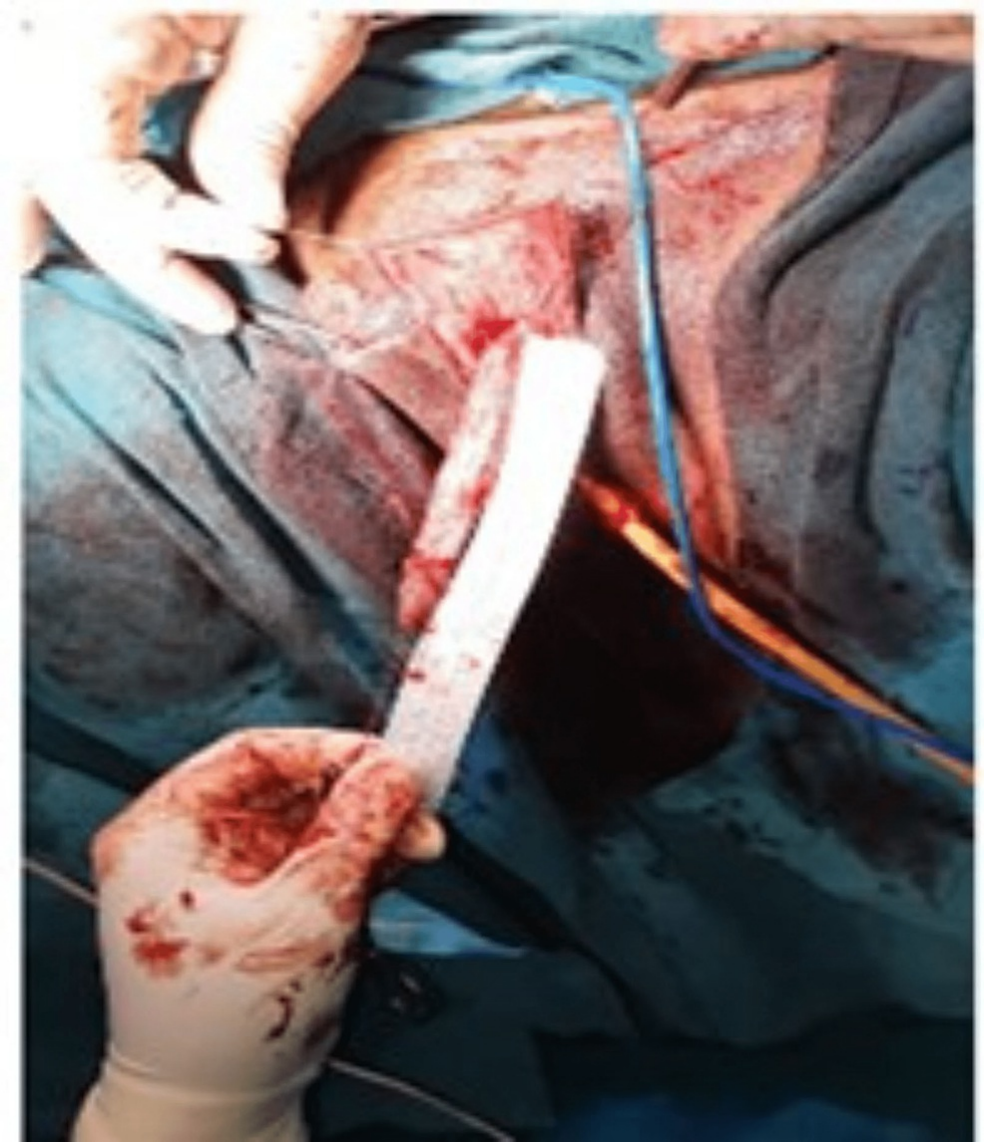
Per-operative picture illustrating clitoromegaly of approximately 9.5 cm. Self-ownership declared.

**Figure 4:**
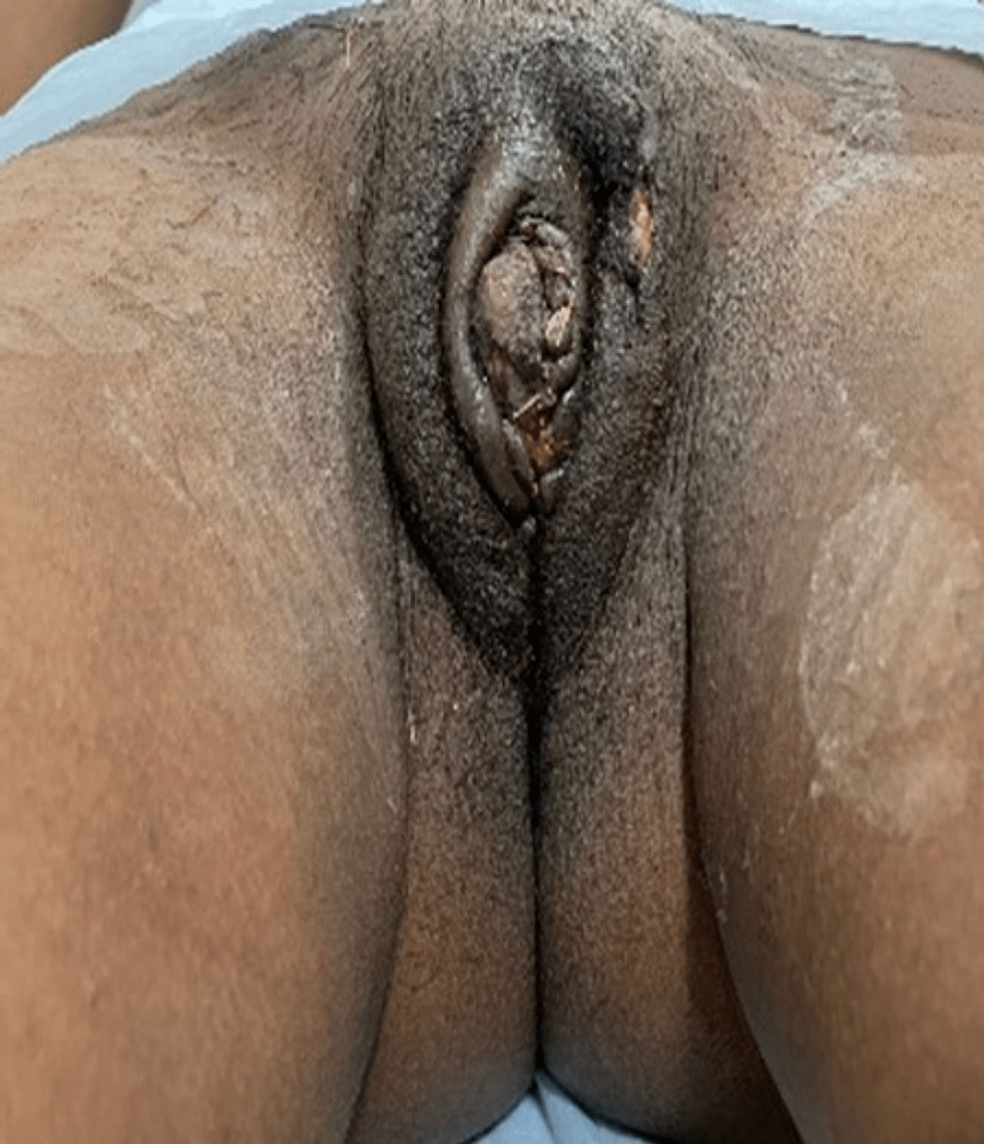
Post-operative day 7. Self-ownership declared.

Following detailed counselling, the patient underwent bilateral gonadectomy with hernia repair and nerve-sparing reduction clitoroplasty. Per-operatively, bilateral gonads were identified, and the total clitoral length was 9.5 cm. Histological examination showed Leydig cell proliferation and atrophic seminiferous tubules with no evidence of any ovarian tissue, hence confirming the gonads as testis. The diagnosis of partial androgen insensitivity was confirmed. Her post-operative stay was uneventful (Figures 1-4).

The repeat hormonal profile after a month showed testosterone level <10ng/dl. The patient was kept on hormonal therapy during her follow-up and planned for augmentation Mammoplasty eventually.

Case report 2

Another 32-year-old married nulligravida appeared in our outpatient department with a six-month history of bilateral inguinal area edoema and pain. She had no notable family history. The patient had primary amenorrhea but had failed to follow up.

**Figure 5:**
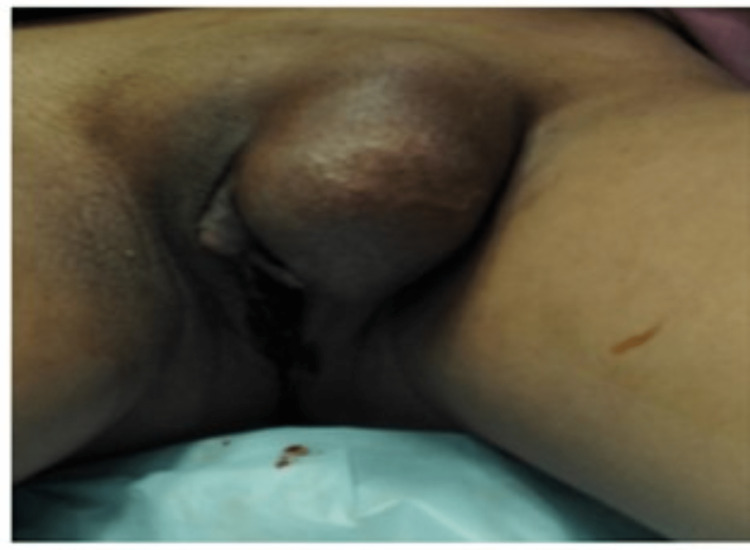
Pre-operative physical characteristics show inguinal hernia with clitoromegaly of 3.0 cm. Self-ownership declared.

**Figure 6:**
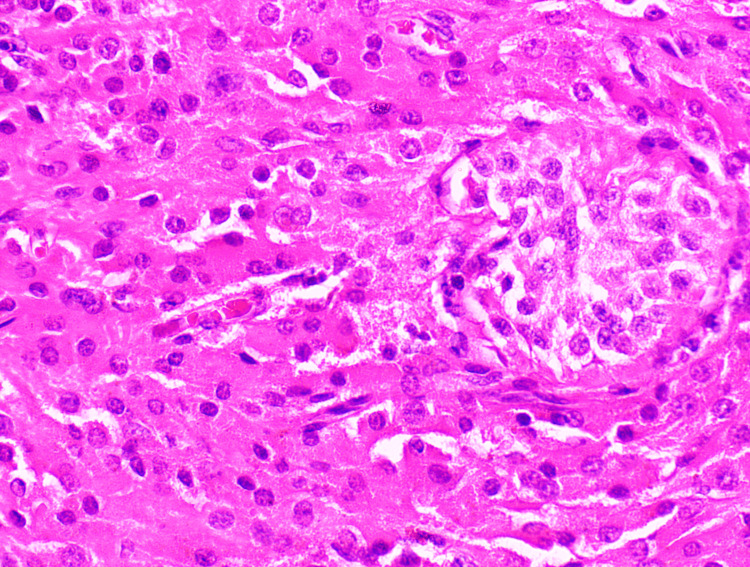
Histopathological slide shows mixed germ cell tumour with atrophied seminiferous tubules. Self-ownership declared.

Her height was 170 cm, her weight was 62 kg, and her BMI was 21.4 kg/m^2^. Tanner stage 4 breast development: axillary hair is present with pubic hair development (Tanner stage 4). On examination, there was bilateral firm tender swelling (6 cm × 4 cm and 4 cm × 4 cm) with a positive cough impulse. She had clitoromegaly of about 3 cm (on the phall-o-meter) with otherwise well-developed female external genitalia and an 8 cm blind vaginal pouch. Her ultrasound revealed non-existent ovaries, uterus, cervix, and testicular-like abnormalities in the inguinal region. Serum testosterone level was 178 ng/dL (typical adult male 200-800 ng/dL, adult female 20-80 ng/dL); other hormone levels were within normal limits. The karyotype was 46XY, with no structural/numerical aberrations, confirming the diagnosis of partial androgen insensitivity. High cord ligation with bilateral orchiectomy was done with a refusal to further any cosmetic surgical intervention. Histopathological examination revealed a mixed germ cell testicular tumour (Figures 5-6).

## Discussion

Androgen insensitivity syndrome is an X-linked inheritance that results in a person who is genetically male (46 XY) but resistant to male hormones (called androgens) and thus has some of the physical attributes of a woman with the genetic composition of a man, with an incidence of one in 130,000 births [1]. It includes a range of conditions ranging from complete to partial insensitivity to testosterone, resulting in complete, partial, and mild forms. The phenotype varies based on the function of the residual androgen receptor, ranging from severe under-masculinization with female-like external genitalia to male-appearing genitalia: complete androgen insensitivity syndrome (CAIS) with predominantly female external genitalia (testosterone has no influence on sexual development), partial androgen insensitivity syndrome (PAIS) with predominantly male/female or ambiguous genitalia (partial response to testosterone), and mild androgen insensitivity syndrome (MAIS) with predominantly male external genitalia. Ambiguity in genitalia is normally recognized at birth; nevertheless, if not diagnosed, some children are raised as males with a small phallus and some breast tissue at puberty. Unlike in our situation, some children with clitoromegaly with or without hypospadias may be raised as girls.

Bilateral (39%) or unilateral (28%) inguinal hernia, a positive family history (21%), a mismatch between amniocentesis-karyotype and phenotypic sex (6%), and primary amenorrhea (6%) are some of the clinical manifestations [2]. In a study conducted in Finland [3], vaginal length was proven to be a valuable clinical tool in screening prepubertal girls for androgen insensitivity syndrome. Early detection is particularly critical since prompt treatments can prevent the development of gonadal cancer. However, because there are no clear and sensitive indicators for early diagnosis of pre-malignant alterations in the gonads, early gonadectomy is still suggested in high-risk populations [4]. Once puberty is reached, preventive gonadectomy and ongoing hormone replacement are indicated to maintain bone health [5].

Surgical and non-surgical aesthetic gynaecologic procedures have redefined the notion of beauty [6]. Gender is a concept of society that is frequently predicated on the idea of a biological binary, but even the best scientific tests cannot - and arguably should not - exactly divide all people into "male" and "female" sexes. We underestimate the diversity of sexuality, as external genitalia, hormone profiles, reproductive anatomy, or chromosomal structure cannot properly characterize it [7]. Studies of cosmetic outcomes after genitoplasty in children with genital atypia showed significant improvement (p<0.001) and no difference in ratings was seen between parents and surgeons [8]. However, satisfaction with post-operative cosmesis does not necessarily equate with functional outcomes later in life.

Individuals who live with a rare disease can face challenges that can be overwhelming. These challenges may include limited information about the disease and the treatment, adjustment to daily activities, social isolation, a wide range of emotions, and economic difficulties. A multidisciplinary team with a psychiatrist and a gynaecologist is required who could counsel about the condition and its prevalence, sexual identity, inability to have childbearing capacity, the need for surgery, regular postoperative follow-up, and hormone supplementation.

Limitations

The patients in the study were consenting adults when they underwent genitoplasty, thus eliminating the factor that makes these surgeries controversial for patients with differences in sex development (DSD): that they are almost always too young to consent to the surgeries that shape their bodies into conformity with the gender assigned at birth and the stereotypical sexualities associated with those genders.

## Conclusions

Surgeries done on patients with partial androgen insensitivity syndrome are not only lifesaving procedures but also help them live life on their own terms. It is a matter of great work and research, as gonadectomy after puberty is still controversial. Also, difficulties in determining the absolute malignancy risk, difficulties with hormone therapy, and a lack of studies supporting different protocols need attention and focus.
